# Cholera in Louisville

**Published:** 1851-09

**Authors:** 


### THE WESTERN JOURNAL.

—

THIRD SERIES.—VOL. VIII.—NO. III.

—

LOUISVILLE, SEPTEMBER 1, 1851.

---

### CHOLERA IN LOUISVILLE.

In our last number, we stated that our city continued to enjoy exemption from all epidemic diseases, notwithstanding the excessively hot weather which had prevailed during a good part of July. We had previously ventured to express the opinion, that we should not this season be visited by cholera in an epidemic form, and gave as the grounds of that belief, that the disease had twice visited us in successive years. On the 4th day of August it was announced that one of the candidates for the Legislature, in the third ward, was ill with cholera, of which, although he lingered beyond the expectation of his physicians, he finally died. An occasional case of the disease was reported from day to day. The hot weather returned, and with it we had drenching thunder showers. Wednesday, the 13th, was spoken of as one of the hottest days of the season, and, though the thermometer did not indicate so high a temperature, it was certainly one of the most sultry and oppressive. Next morning there was a report of several cases of cholera on Market street, and on 4th and 5th streets, near Market, in the very centre of the city; and before night seven or eight persons had died. In the course of the day the streets of the city were two or three times flooded with rain, but during the following night it cleared off and became cooler. The day succeeding, the number of deaths amounted, probably, to ten or twelve, but there was evidently no increase of the disease, most of the attacks having been experienced the previous day. On Saturday, a few new cases continued to be announced in the infected district, and scattering cases were reported in different parts of the city, but still the complaint manifested but little epidemic tendency. There was no increase on Sunday, notwithstanding the return of the hot, sultry weather, which was again accompanied by much thunder and lightning and profuse showers of rain, and on Monday we heard of no new cases of the disease.

Several facts connected with this sudden, local irruption of cholera are noteworthy; and first the character of the weather in which it occurred. No one who was in Lexington in 1833 could fail to be struck with the similarity of the two seasons—the protracted drought, succeeded by repeated, heavy rains, producing an atmosphere saturated with moisture. It may be also worth remarking, that the prevailing wind during this unseasonable weather was from the north-east.

Another most impressive fact is, that the cases which occurred in the district referred to, with hardly a solitary exception, proved fatal. A number of persons doing business in that quarter of the city and a number sleeping there were attacked in other and healthy parts, or carried into them soon after being attacked; but the result was the same—nearly every one died. While this was the case in the infected region, we heard in other quarters of several cases of recovery, in which the first symptoms seemed quite as threatening. It is humbling to the pride of our art to be obliged to add, that this fatality was in no wise attributable to the bad constitutions and bad habits, nor to the neglect of the victims to apply for timely aid. In a few of the first cases no doubt there was delay, for cholera was not suspected; but after the first hours of the morning people and citizens were on the alert and the earliest premonitions of the disorder were regarded. We heard of instances in which physicians were in the house and prescribed for their patients before yet they appeared to be ill, and still the disease marched on to a fatal termination as if nothing had been done to arrest its progress.

The attacks were confined to no age or condition. Two or three lads died, and a number of young men and women. We saw a middle-aged citizen a few hours after he had been seized; he was already in collapse and pulseless, and died three hours afterwards. He was an Irishman, and though not a stout man was apparently in good health the day before this attack. While he was sinking, an Englishman, of about the same age, of robust frame, who, when first seized, said he had never before been sick in his life, was dying in his neighborhood.

It was the common remark of the physicians that the prostration in all the fatal cases was quite out of proportion to the alvine evacuations, not a few patients dying after a few comparatively trifling discharges. One of the cases to which we have just alluded was of this description; the patient vomited once or twice, had two or three inconsiderable discharges from his bowels, and was in collapse. On the other hand, patients in other quarters of the city had the most profuse evacuations and recovered. Dr. P. Cochran mentioned to us a case which resulted favorably after the patient, at a single discharge, had filled his chamber-pot so full that it ran over upon the floor. In the former case, the subjects seemed to be asphyxiated by some subtile poison; they were the subjects of genuine cholera *asphyxia.* Contemporaneously with these malignant cases, attacks of ordinary cholera morbus were occurring about town, which physicians found easily manageable and without any tendency to assume a malignant form. Even when neglected and the alvine dejections were copious, there was still no approach nor disposition to collapse in these cases.

In a number of instances, more than one fatal case occurred in the same house and family, but no where beyond the limits of the pestilent district. Not an instance has come under observation in which the disease appeared to be transported.

It is certainly a remarkable and interesting fact, that the disease which sprung up so suddenly and with so much malignity, and, we may add, without any visible local cause, did not manifest any tendency to spread, but after a day or two began to decline when scarcely a single inhabitant of the sickly region had deserted his house. In some cases, the sudden cessation of the epidemic has been ascribed to the flight of the citizens; but here there was no such cause to account for it. Business has continued to be transacted, and the people of that district have eaten and slept in their houses as if no pestilence had seemed to impend over them.

Circumstances still indicate, we think, that Louisville will escape a visitation of epidemic cholera. The disease, with much that is inscrutable, exhibits great constancy of character as respects its progress. While we write, we hear of its appearance in Versailles, two years after its visit to Lexington; just as was the case when it was epidemic in our country before. On three different occasions it broke out in Louis ville during its former reign, but never became epidemic. Returning again and again it was still very much confined, in each irruption, to certain circumscribed localities. We have now been visited by it three successive years. In 1849 its prevalence was more general; last summer it broke out, with alarming malignity, first in the lower and then in the upper part of the city, but subsided in a short time without becoming epidemic. In its late descent, as we have seen, its ravages, though frightful, were restricted to an area bounded by a few squares. If it is to conform here as it has done so generally elsewhere to its former course, it ought now soon to disappear from Louisville without becoming epidemic.

Bearing upon the portability of the cause of cholera, these facts are curious—that a man who had been on the river and in Louisville during this out-break of the disease, went into the country near Charlestown, Ind., sickened and died; that two members of his family were also seized with cholera and died, and two or three relatives who visited them also contracted the disease and fell victims to it; while at the same time the hundreds of persons who were in daily and hourly intercourse with the sick in Louisville failed to carry the infection beyond the striken district. Not a few of the friends who waited on the sick and dying contracted the malady and died in various parts of the city, but in no instance was the infection spread by them. It can hardly be said, that it was because our city was free from all the causes generally esteemed favorable to the propagation of cholera, that the disease did not become epidemic. So far from this being the case, we doubt whether our streets ever afforded the conditions of disease in a higher degree—"The offence is rank and smells to heaven," was the remark and the feeling of everybody. Nor can the favorable issue be justly set down to the weather. Just the very meteorological conditions had obtained which are reputed the best calculated to excite epidemic cholera, and though there was a favorable change of weather the day after the irruption of the disease, the clear, bracing air did not prevail long. On Sunday the rains returned, and the streets of the city were again flooded, the air at the same time being hot and sultry. Notwithstanding, the number of cases continued rapidly to diminish, and during that week, from the best information we have been able to obtain, only two or three people died of cholera. We conclude therefore, that while it would be illogical to say that the weather and foul streets have nothing to do with cholera, cholera may abate in the midst of dirty streets and the worst sort of weather.

